# Objectively Measured Sedentary Time Is Related to Quality of Life among Cancer Survivors

**DOI:** 10.1371/journal.pone.0087937

**Published:** 2014-02-05

**Authors:** Stephanie M. George, Catherine M. Alfano, Jay Groves, Zafer Karabulut, Kirsten L. Haman, Barbara A. Murphy, Charles E. Matthews

**Affiliations:** 1 Applied Research Program, Division of Cancer Control and Population Sciences, National Cancer Institute, Bethesda, Maryland, United States of America; 2 Office of Cancer Survivorship, National Cancer Institute, Bethesda, Maryland, United States of America; 3 Vanderbilt Dayani Center for Health and Wellness, Vanderbilt University Medical Center, Nashville, Tennessee, United States of America; 4 Department of Psychiatry, Vanderbilt University School of Medicine, Nashville, Tennessee, United States of America; 5 Division of Hematology/Oncology, Department of Medicine, Vanderbilt University, Nashville, Tennessee, United States of America; 6 Nutritional Epidemiology Branch, Division of Cancer Epidemiology and Genetics, National Cancer Institute, Bethesda, Maryland, United States of America; The James Cook University Hospital, United Kingdom

## Abstract

**Purpose:**

While exercise has been shown to be beneficial in improving health-related quality of life (HRQOL) among cancer survivors, evidence is limited on the independent role of sedentary behavior. We examined how objectively measured sedentary time was associated with HRQOL among long-term cancer survivors.

**Methods:**

This cross-sectional study included 54 cancer survivors, on average 3.4 years postdiagnosis, who were enrolled into an exercise trial designed to improve cognitive function. At baseline, we measured sedentary time and moderate-vigorous intensity physical activity with the ActivPal, cardiorespiratory fitness with treadmill testing, and self-reported HRQOL with an established scale (SF-36). In multivariate models, we regressed HRQOL on sedentary time (percent of waking time spent sitting and lying).

**Results:**

Survivors with higher sedentary time had significantly poorer physical functioning (β = −0.50, p = 0.028), general health (β = −0.75, ptrend = 0.004), and physical summary scores (β = −0.34, p = 0.003). We did not observe associations between sedentary time and role-physical (p = 0.342), bodily-pain (p = 0.117), vitality (p = 0.095), social functioning (p = 0.407), role-emotional (p = 0.509), mental health (p = 0.494), or mental summary scores (p = 0.527).

**Conclusion:**

In this cross-sectional study of cancer survivors, we observed deleterious associations between sedentary time and aspects of physical HRQOL. Future prospective studies of sedentary time and HRQOL are needed to establish temporality and to facilitate the design of effective health promotion interventions for cancer survivors.

## Introduction

It is estimated that over 14 million cancer survivors are living in the US today, and that this number will rise to 18 million by the year 2022 [1]. Worldwide is it estimated there are 28 million cancer survivors within 5 years of diagnosis [2]. Survivors face many physical and emotional challenges throughout their treatment and recovery, including persistent and profound adverse effects on physical and mental quality of life [3] which compromise survivors' abilities to maintain independent lifestyles. Even five years after diagnosis, many survivors still experience impaired physical functioning [4], [5], and the importance of preserving physical function within this expanding population has grown exponentially [6].

A recent roundtable by the American College of Sports Medicine concluded that exercise is safe during and after cancer treatment and results in improvements in health-related quality of life (HRQOL) [7]. Sedentary behavior —common behavior involving prolonged sitting or reclining that requires only low levels of energy expenditure— has been shown to have deleterious health consequences independent of the beneficial effects of exercise in general adult populations [8]. Little is known about the relationship between sedentary behavior and HRQOL among survivors. One study on this topic found that self-reported television watching time was inversely related to functional well-being among colorectal cancer survivors [9], but another found that self-reported sedentary time was not related to HRQOL among breast cancer survivors [10]. Because sedentary behavior can be feasibly modified in adults [11], there is a need for more research in this area to inform behavioral interventions for survivors.

The purpose of this study was to evaluate how objectively measured sedentary time was related to HRQOL among long-term cancer survivors.

## Methods

### Participants

The present cross-sectional study was conducted among cancer survivors recruited into the Activity Trial for Improving Chemobrain (TACTIC), a study designed to evaluate the effect of a six-month exercise intervention on cognitive functioning after chemotherapy. Main findings from the trial have not yet been published. Cancer survivors were recruited via the Vanderbilt Ingram Cancer Center (VICC) cancer registry, VICC oncology clinics, a press release from VICC to local media outlets, mass emails via Medical Center Communications to all Vanderbilt faculty and staff, and mass emails to targeted participants in the Clinical Research Volunteer Registry of the General Clinical Research Center, local oncology clinics, and cancer support groups and services. To be eligible for the trial, all survivors needed to have completed at least four rounds of chemotherapy in the past five years, have no evidence of disease, and to have reported the onset of persistent cognitive difficulties following chemotherapy. In addition, survivors included had no prior diagnosis of cancer of the central nervous system, did not engage in regular exercise in the past year (i.e., > = 5 days/wk, > = 20 min/d, > = 3 months), did not have cardiovascular disease, orthopedic problems, or medical conditions that could be worsened by exercise, be 18 years of age or older, and not be pregnant at the start of the study. We obtained written informed consent from all study participants. The study was approved by the institutional review board at Vanderbilt, in accord with assurances filed with and approved by the US Department of Health and Human Services. Of the 64 survivors in this intervention, we excluded from our analysis those with missing data for sedentary time (n = 1), fitness (n = 1), HRQOL (n = 6), and radiation (n = 2). Our final sample size was 54.

### Measurements

#### Sedentary behavior and physical activity

We assessed time during the waking day spent in sedentary behavior and moderate-vigorous intensity physical activity (MVPA) using the activPAL device (PAL Technologies, Glasgow, Scotland). The monitor is worn on the mid-right thigh, and uses information about thigh position to estimate time spent in different body positions (horizontal = lying or sitting; vertical = standing or stepping). The instrument records the start and stop time of each individual bout (or event) of lying or sitting, standing, and stepping. Participants wore the device during waking hours, exclusive of bathing and swimming, for seven consecutive days. They were asked to record the time they got out of/into bed and the times they wore the monitor each day. Sedentary behavior was measured as time spent sitting or lying *during the waking day*, and physically active behavior was measured as the sum of time spent standing or stepping. For each participant, we calculated percent of day spent in sedentary behavior (sedentary behavior/wear time). The device also estimates the energy cost of ambulatory activities using a prediction equation that employs stepping cadence and duration as the predictor variables (MET-hours = (1.4×duration [hours])+(4–1.4)×(cadence [steps/minute]/120)×duration activPAL. We calculated time recorded in moderate-vigorous stepping activities (i.e., MVPA, 3+ METs) using these data. ActivPAL accuracy for measuring body posture in laboratory settings is 95 to 100% [12], and there is also good agreement for sedentary time (R^2^>0.94) between activPAL and direct observation in a free-living studies [13], [14]. We are unaware of validation studies for MVPA estimates from activPAL, but the duration estimates derived using this approach compare favorably to the 1952 count per minute ActiGraph threshold of Freedson [14]. Quality control checks were implemented to identify non-compliance during the wearing period (i.e., <10 hrs/d of wear) or other problems with the data (i.e., monitor malfunction or wearing the device upside down). Days with at least 10 hours of wearing were considered valid and were used to create daily averages of relevant parameters. The average number of hours the monitor was worn was 15.4 (SD = 2.1) hours/day and the average number of days monitored was 5.8 (SD = 1.9).

#### HRQOL

HRQOL was assessed using the Medical Outcomes Study SF-36 Quality of Life Scale [15], a 36 item, valid and reliable short-form instrument [16], [17] that is widely used among medically ill and healthy populations [18]. The SF36® yields eight subscales (physical functioning, role-physical, bodily pain, general health, social functioning, role-emotional, mental health, vitality) and two component summary scores (physical and mental). For all scales, a higher score (0–100) represents better functioning and well-being. The component summary scores were standardized on a T-score metric, with a score of 50 representing the U.S. general population average (standard deviation = 10).

#### Fitness

Cardiorespiratory fitness was measured at baseline by medically supervised symptom-limited maximal treadmill testing using a modified Bruce protocol starting at 1.7 mph and 0 percent grade [19]. The equation of Foster and colleagues [20] was used to estimate peak fitness levels in terms of METs by dividing the predicted values by 3.5 ml/kg/min. All testing was completed using the established protocols at the Vanderbilt Dayani Center for Health and Wellness.

#### Other covariates

At baseline, trained study personnel collected anthropometric measures (height, weight, skinfolds) and body composition via bioelectrical impedance [21]. Participants reported on their demographic characteristics. We abstracted from medical records information regarding cancer diagnoses (diagnosis date, type, tumor stage) and treatment (dates, types), including receipt of radiation treatment in addition to chemotherapy.

### Statistical Analysis

Differences in descriptive characteristics of women with higher vs. lower sedentary time were tested using t-tests (continuous variables) or likelihood ratio chi-square tests (categorical variables).

We regressed HRQOL linearly on sedentary time in multivariate models. To identify potential confounders in our analysis, we evaluated all covariates listed in Table 1 (age, sex, MVPA, cardiorespiratory fitness, BMI, cancer type and stage, radiation treatment, and years since treatment) individually in relation to the base HRQOL and sedentary time model. Factors that changed the magnitude of beta values for the physical or mental HRQOL summary scores by at least 10%, improved model fit by comparison of log likelihood values in full and reduced models and/or were statistically significant were retained in our final model. Our final model for analyses of sedentary time included age, MVPA, fitness, and radiation. BMI did not meet the criteria for model inclusion and additional adjustment for BMI did not result in substantial changes to the estimates obtained.

**Table 1:** Demographic, Clinical, and Lifestyle Characteristics of Cancer Survivors in TACTIC.

Characteristic	Mean (SE)	N	%
Age	54.3 (8.8)		
ActivPal wear time (hrs/day)	15.4 (2.2)		
% Daily sedentary time^1^	69.2 (10.9)		
% Daily standing time^1^	20.5 (8.5)		
% Daily stepping time^1^	10.3 (4.1)		
Hours per day spent sitting or lying (activPAL)	10.7 (2.3)		
Hours per day spent in moderate-vigorous physical activity (activPAL)	0.5 (0.2)		
Cardiorespiratory fitness level (METs, treadmill test)	6.0 (1.4)		
Body mass index (kg/m^2^)	28.7 (6.9)		
Sex			
Men		9	17
Women		45	83
Cancer stage			
I		17	31
II		22	41
III		10	19
Not reported		5	9
Cancer type			
Breast		36	67
Colorectal, hematological, head and neck, lung, cervical		18	33
Radiation treatment in addition to chemotherapy			
Yes		33	61
No		21	39

1 calculated using activPAL as % of waking day spent in that behavior.

## Results

On average, cancer survivors spent the majority (69%) of their 15.4 hour waking day engaged in sedentary behaviors (**Table 1**), and they spent roughly 21% of their time standing, and 11% of their time stepping. Within the stepping time, participants spent an average of 0.5 hours per day in MVPA. About half of the participants engaged in 30 minutes of MVPA a day and may have been meeting current guidelines for physical activity (data not shown).

As shown in Table 2, survivors with higher sedentary time reported significantly poorer physical well-being, as indicated by lower scores on several physical HRQOL indices, including overall physical summary scores (β = −0.34, p = 0.003), and subscores for physical functioning (β = −0.50, p = 0.028)and general health (β = −0.75, p = 0.004), and we did not observe associations between sedentary time and role-physical (p = 0.342), bodily-pain (p = 0.117), vitality (p = 0.095), social functioning (p = 0.407), role-emotional (p = 0.509), mental health (p = 0.494), or mental summary scores (p = 0.527).

**Table 2:** Multivariate associations^1^ between objectively measured sedentary time (continuous) and HRQOL scores.

	β	se	p-value
Physical summary score	−0.34	0.11	0.003
Physical functioning	−0.50	0.22	0.028
Role-physical	−0.50	0.52	0.342
Bodily pain	−0.39	0.24	0.117
General health	−0.75	0.25	0.004
Mental summary score	0.09	0.14	0.527
Vitality	−0.53	0.31	0.095
Social functioning	−0.27	0.32	0.407
Role-emotional	0.37	0.56	0.509
Mental health	0.15	0.22	0.494

1 Beta values and standard errors from linear regression models adjusted for age, radiation treatment, hrs/day moderate-vigorous intensity physical activity (activPAL), and cardiorespiratory fitness.

## Discussion

Our results fill a gap in the literature by demonstrating, for the first time, the relationship of objectively measured sedentary time and HRQOL among long-term cancer survivors. The only other studies examining sedentary behavior and HRQOL among cancer survivors used self-report measures of sedentary time; one reported a deleterious association [9] and the other reported no association [10]. The present study used an objective measure vs. a self-reported measure of sedentary time, and we found that sedentary time was significantly related to overall physical HRQOL, perhaps specifically for physical functioning and general health.

It is biologically plausible that sedentary time may compromise physical HRQOL. Sedentary behavior is thought to displace time otherwise spent in the light-intensity non-exercise activities of daily living [8], and because these activities result in a substantial amount of physical activity energy expenditure [22], [23], [24] and ambulation, they may be important in the preservation of physical health after cancer. Prolonged sedentary time has been linked to disruptions of metabolic activity within individual muscle cells, insulin response, oxidative stress, and DNA repair mechanisms [25], and these mechanisms may be important to physical HRQOL.

Self-reported sedentary behavior has been shown to be higher in cancer survivors than individuals without cancer [26], stressing the importance of this behavior as a target for health promotion efforts in this group. In fact, current national physical activity guidelines for adults include statements about avoiding inactivity [27] and limiting discretionary screen/sedentary time [28]. As assessed by the activPAL, cancer survivors spent 69% of their 15.4 hour waking day sitting and lying down, which calculates to about ∼10.6 hours of daily sedentary time. This estimate of % sedentary time is consistent and almost identical to national survey estimates among breast [29] and prostate [30] cancer survivors.

It is important to understand these findings in the context of what constitutes a the minimally important difference (MID) in the SF-36 necessary that would signify a meaningful or clinical effect [31] Cohen's (1992) criteria has been used previously in cancer populations [32] and suggests that a small effect is indicated by a 0.20 SD and a medium effect by a 0.50 SD [33], there is support in the literature that MIDs in HRQOL fall within this range [34], [35]. Applying this criterion to our study, a small and medium effect would translate to a 2 and 5 point difference. Extrapolating the results of our study, every 10% increase in sedentary time (∼every 92 minute increase) was associated with a statistically significant 3.5 point decrease in physical component summary scores, a 5 point decrease in physical functioning, scores and a 7.5 point decrease in general health scores, suggesting that these differences may have clinical relevance.

Our study had several strengths. The use of objective measures of physically active and sedentary behaviors and cardiorespiratory fitness negated the need for retrospective recall of activities. Our measurements were also made about 3.4 years after chemotherapy was completed, so it is unlikely that cancer treatment would have affected health behaviors or outcomes. In addition, we had information on cancer diagnoses and treatment from medical records for all survivors. Further, all participants in the present study, aside from their previous cancer diagnosis and treatment experience, were free of major chronic diseases or orthopedic limitations, were suitable to participate in an unsupervised moderate intensity exercise program, and thus were a homogenous group with respect to physical health.

However, this study had several limitations. Given the present study's cross-sectional design, we were not able to establish temporality of the sedentary time-HRQOL relationship. Further, our results should be interpreted in the context of our study population's small size and cancer diagnoses our findings may be most applicable to cancer survivors that received chemotherapy and survived at least 3 years after their treatment and therefore may be less applicable to other groups of survivors. Additional research is needed to confirm the present results and determine the extent to which these findings are applicable to other populations. Our study also focused only on objectively measured sedentary behavior and HRQOL at one point during the cancer survivorship period (∼3.4 years post-treatment), and because the effect of sedentary time may vary during and after cancer treatment, more research is needed on sedentary time and change in sedentary time at different points during survivorship. It would also be of interest in future research to also explore how postdiagnosis sedentary time is related to preservation of HRQOL, given recent findings documenting an independent association of sedentary and decline in physical function in postmenopausal women without cancer [36].

Given that we found associations between sedentary time and aspects of physical HRQOL even after adjustment for MVPA, our study provides preliminary evidence that sedentary behavior may be another independent health behavior, in addition to MVPA participation, which could be targeted and changed to preserve functioning after cancer. There are a multitude of opportunities to address this behavior, both in the clinic and home settings, and an intervention targeting the interruption of sedentary behavior in cancer survivors is being evaluated [37], [38], though it only utilizes a 5 question self-report measure of sedentary behavior. Future prospective studies with objective and validated self-report measures of sedentary time and HRQOL are needed to establish temporality, evaluate the potential dose-response relationship, and facilitate the design of effective interventions to promote health, preserve function, and ultimately reduce cancer comorbidities in this growing population.
